# Tobacco smoking is associated with methylation of genes related to coronary artery disease

**DOI:** 10.1186/s13148-015-0088-y

**Published:** 2015-05-14

**Authors:** Rebecca V Steenaard, Symen Ligthart, Lisette Stolk, Marjolein J Peters, Joyce B van Meurs, Andre G Uitterlinden, Albert Hofman, Oscar H Franco, Abbas Dehghan

**Affiliations:** Department of Epidemiology, Erasmus University Medical Centre, ‘s Gravendijkwal 230, 3015 CE Rotterdam, The Netherlands; Netherlands Institute for Health Sciences, Erasmus University Medical Centre, ‘s Gravendijkwal 230, 3015 CE Rotterdam, The Netherlands; Department of Internal Medicine, Erasmus University Medical Centre, ‘s Gravendijkwal 230, 3015 CE Rotterdam, The Netherlands; The Netherlands Genomics Initiative-sponsored Netherlands Consortium for Healthy Aging (NGI-NCHA), Einthovenweg 20, 2333 ZC Leiden/ ‘s Gravendijkwal 230, 3015 CE Rotterdam, The Netherlands

**Keywords:** DNA methylation, mRNA expression, Tobacco smoking, Coronary artery disease, White blood cells

## Abstract

**Background:**

Tobacco smoking, a risk factor for coronary artery disease (CAD), is known to modify DNA methylation. We hypothesized that tobacco smoking modifies methylation of the genes identified for CAD by genome-wide association study (GWAS).

**Results:**

We selected genomic regions based on 150 single-nucleotide polymorphisms (SNPs) identified in the largest GWAS on CAD. We investigated the association between current smoking and the CpG sites within and near these CAD-related genes. Methylation was measured with the Illumina Human Methylation 450K array in whole blood of 724 Caucasian subjects from the Rotterdam Study, a Dutch population based cohort study.

A total of 3669 CpG sites within 169 CAD-related genes were studied for association with current compared to never smoking. Fifteen CpG sites were significantly associated after correction for multiple testing (Bonferroni-corrected *p* value <1.4 × 10^−5^). These sites were located in the genes *TERT*, *SARS*, *GNGT2*, *SMG6*, *SKI*, *TOM1L2*, *SIPA1*, *MRAS*, *CDKN1A*, *LRRC2*, *FES* and *RPH3A*. In 12 sites, current smoking was associated with a 1.2 to 2.4 % lower methylation compared to never smoking; and in three sites, it was associated with a 1.2 to 1.8 % higher methylation. The effect estimates were lower in 10 of the 15 CpG sites when comparing current to former smoking. One CpG site, cg05603985 (*SKI*), was found to be associated with expression of nearby CAD-related gene *PRKCZ*.

**Conclusions:**

Our study suggests an effect of tobacco smoking on DNA methylation of CAD-related genes and thus provides novel insights in the pathways that link tobacco smoking to risk of CAD.

**Electronic supplementary material:**

The online version of this article (doi:10.1186/s13148-015-0088-y) contains supplementary material, which is available to authorized users.

## Background

In recent years, large genome-wide association studies (GWAS) have been conducted to identify genetic risk factors for a vast amount of diseases including coronary artery disease (CAD). These GWAS have successfully identified tens of single-nucleotide polymorphisms (SNPs) located in genes and their vicinity that might play a role in the pathophysiology of CAD. The CARDIoGRAMplusC4D consortium is the largest CAD-GWAS consortium comprising 63,746 CAD cases and 130,681 controls [1]. This consortium has found 46 susceptibility loci significantly associated with the risk of CAD and 104 loci suggestive for CAD (FDR < 0.05).

One of the major risk factors for CAD is tobacco smoking which accounts for 10–15 % of the risk [2]. Recent studies have shown that smoking can interact with genetic variation to increase the risk of CAD [3, 4]. One of the potential mechanisms for this interaction is DNA methylation. DNA methylation is the attachment of a methyl group to a nucleotide which occurs most often at the cytosine nucleotide of CpG dinucleotides. Methylation has varying functions at different locations in the human genome including influence on gene expression [5].

Since studies have established an important role for smoking in DNA methylation [6–8], we hypothesized that tobacco smoking changes DNA methylation of genes in/near loci identified for CAD, which in turn could alter gene expression of these genes. We therefore investigated the association between DNA methylation of genes in/near CAD-GWAS loci in whole blood and tobacco smoking in the Rotterdam Study. Furthermore, we investigated the association between methylation and expression of CAD-related genes nearby the identified differentially methylated sites.

## Results

Characteristics of the participants under study are summarized in Table 1. Of the 724 subjects in the study, 195 were current smokers and 201 were never smokers. The mean age was 59.9 years. Among the current smokers, 50 % was male, among the never smoker 37 % (*p* = 0.008).

**Table 1:** Characteristics of study population

	Total	Current smokers	Former smokers	Never smokers	*p*-value^a^
N	724	195	319	210	
Age (years)	59.9 (8.2)	58.1 (6.7)	61.5 (8.6)	59.2 (8.4)	0.15
Sex (male)	334 (46 %)	98 (50 %)	155 (49 %)	78 (37 %)	0.008
BMI (kg/m^2^)	27.6 (4.6)	26.8 (4.3)	28.0 (4.5)	27.7 (4.9)	0.05
Proportion CD8+ T-cells^b^	0.09 (0.05)	0.10 (0.05)	0.09 (0.06)	0.10 (0.05)	0.81
Proportion CD4+ T-cells^b^	0.26 (0.08)	0.27 (0.07)	0.26 (0.08)	0.26 (0.08)	0.33
Proportion NK cells^b^	0.14 (0.06)	0.11 (0.06)	0.14 (0.06)	0.14 (0.06)	<0.001
Proportion B-cells^b^	0.10 (0.04)	0.10 (0.04)	0.10 (0.03)	0.10 (0.04)	0.59
Proportion monocytes^b^	0.08 (0.03)	0.08 (0.03)	0.08 (0.03)	0.08 (0.03)	0.55
Proportion granulocytes^b^	0.37 (0.11)	0.38 (0.11)	0.37 (0.12)	0.37 (0.11)	0.12
Nr. erythrocytes (10^9^/L)	4.90 (0.37)	4.86 (0.36)	4.93 (0.37)	4.88 (0.38)	0.67
Nr. platelets (10^9^/L)	283.8 (63.9)	293.2 (69.3)	280.2 (61.0)	280.4 (62.4)	0.05
Fasting (yes)^c^	717 (99 %)	192 (99 %)	315 (99 %)	210 (100 %)	0.07
Alcohol (g/day)	18.2 (11.3)	19.3 (12.8)	18.9 (11.5)	16.0 (8.9)	0.002
RNA quality score	8.38 (0.51)	8.36 (0.50)	8.39 (0.49)	8.38 (0.54)	0.66
Type 2 diabetes mellitus	53 (7.3 %)	7 (3.6 %)	34 (10.6 %)	12 (5.7 %)	0.31
Total cholesterol (mmol/L)	5.6 (1.3)	5.6 (1.1)	5.6 (1.5)	5.6 (1.1)	0.88
Triglycerides (mmol/L)	1.49 (0.87)	1.68 (1.14)	1.43 (0.68)	1.39 (0.80)	0.005
Systolic blood pressure (mmHg)	135.7 (61.7)	132.3 (61.7)	137.5 (64.3)	136.3 (57.6)	0.50
Diastolic blood pressure (mmHg)	84.9 (63.3)	83.5 (61.7)	85.6 (66.7)	85.1 (59.7)	0.79

Data are mean (SD) or *n* (%)

^a^Current versus never

^b^Houseman-estimated white blood cell proportions

^c^The subjects who provided blood in fasting condition

The 150 SNPs identified in the CAD GWAS were annotated to 85 genes with an in-gene variant and 84 cis-expression-quantitative trait loci (cis-eQTL) within 1 Mb of the identified SNPs as found in a large publically available blood cis-eQTL database (FDR < 0.05) (Additional file 1: Table S1) [9]. These genes had 3669 methylation sites measured on the array within and near the gene as provided by Illumina (Additional file 1: Table S2). After correction for multiple testing, 15 CpG sites were significantly associated with current smoking (*p* < 1.4 × 10^−5^) (Table 2). Current tobacco smoking was associated with a 1.2 to 2.4 % lower DNA methylation compared to never smoking in 12 of the CpG sites. In three CpG sites, current tobacco smoking was associated with a 1.2 to 1.8 % higher DNA methylation. The effect estimates of the associations were robust to further adjustment for total cholesterol, triglyceride levels, systolic blood pressure, daily alcohol intake and type 2 diabetes mellitus as potential confounders or mediators. In a sensitivity analysis in current smokers, two sites were significantly associated with cumulative exposure to tobacco smoking (Table 3).

**Table 2:** Significant associations between tobacco smoking and methylation of CAD genes

	Current-Never		Adjusted model		Current-Former		Adjusted model	
CpG site	Estimate (se)	*p*-value	Estimate (se)	*p*-value	Estimate (se)	*p*-value	Estimate (se)	*p*-value
cg24908166	−0.014 (0.003)	1.1 × 10^−7^	−0.015 (0.003)	1.6 × 10^−8^	−0.006 (0.002)	0.02	−0.007 (0.003)	0.01
cg12324353	−0.012 (0.003)	1.3 × 10^−7^	−0.012 (0.002)	3.5 × 10^−7^	−0.007 (0.002)	2.8 × 10^−3^	−0.007 (0.002)	2.1 × 10^−3^
cg03725309	−0.024 (0.005)	2.0 × 10^−7^	−0.022 (0.005)	3.8 × 10^−6^	−0.017 (0.004)	4.2 × 10^−5^	−0.016 (0.004)	1.2 × 10^−4^
cg00980784	−0.015 (0.003)	2.5 × 10^−7^	−0.014 (0.003)	3.5 × 10^−6^	−0.015 (0.003)	2.4 × 10^−8^	−0.015 (0.003)	8.6 × 10^−8^
cg13916835	−0.021 (0.004)	3.0 × 10^−7^	−0.021 (0.004)	1.3 × 10^−6^	−0.013 (0.004)	1.5 × 10^−3^	−0.013 (0.004)	1.9 × 10^−3^
cg09469355	−0.017 (0.003)	7.7 × 10^−7^	−0.017 (0.003)	8.5 × 10^−7^	−0.015 (0.003)	3.8 × 10^−6^	−0.015 (0.003)	1.6 × 10^−6^
cg05603985	−0.014 (0.003)	8.4 × 10^−7^	−0.013 (0.003)	3.6 × 10^−5^	−0.014 (0.003)	1.3 × 10^−7^	−0.014 (0.003)	2.4 × 10^−7^
cg04324276	−0.015 (0.003)	3.2 × 10^−6^	−0.015 (0.003)	1.2 × 10^−5^	−0.010 (0.003)	2.4 × 10^−4^	−0.010 (0.003)	5.2 × 10^−4^
cg25468516	−0.015 (0.003)	3.6 × 10^−6^	−0.014 (0.003)	3.0 × 10^−5^	0.011 (0.003)	2.3 × 10^−4^	0.012 (0.003)	1.2 × 10^−4^
cg22907952	−0.011 (0.002)	4.5 × 10^−6^	−0.010 (0.003)	6.9 × 10^−5^	−0.007 (0.002)	1.1 × 10^−3^	−0.007 (0.002)	1.5 × 10^−3^
cg15474579	−0.017 (0.004)	5.0 × 10^−6^	−0.013 (0.004)	5.2 × 10^−4^	−0.016 (0.003)	2.0 × 10^−6^	−0.016 (0.003)	7.6 × 10^−6^
cg20496896	0.012 (0.003)	6.7 × 10^−6^	0.013 (0.003)	4.6 × 10^−6^	0.005 (0.002)	0.06	0.005 (0.002)	0.03
cg09397246	0.018 (0.004)	1.0 × 10^−5^	0.018 (0.004)	3.1 × 10^−5^	0.017 (0.003)	5.6 × 10^−7^	0.017 (0.003)	1.9 × 10^−6^
cg26405020	0.014 (0.003)	1.2 × 10^−5^	0.014 (0.003)	1.4 × 10^−5^	0.008 (0.003)	2.6 × 10^−3^	0.007 (0.003)	8.2 × 10^−3^
cg18236066	−0.014 (0.003)	1.3 × 10^−5^	−0.015 (0.003)	2.5 × 10^−5^	−0.011 (0.003)	1.4 × 10^−4^	−0.011 (0.003)	1.8 × 10^−4^

Bonferroni-corrected threshold 1.4 × 10^−5^

Current-Never: adjusted for age, sex, BMI, Houseman estimates, batch effects

Adjusted model: current-never, adjusted for age, sex, BMI, Houseman estimates, batch effects, systolic blood pressure, total cholesterol, triglycerides, daily alcohol intake, type 2 diabetes mellitus

Current-Former: adjusted for age, sex, BMI, Houseman estimates, batch effects

Adjusted model: current-former, adjusted for age, sex, BMI, Houseman estimates, batch effects, systolic blood pressure, total cholesterol, triglycerides, daily alcohol intake, type 2 diabetes mellitus

**Table 3:** Association between packyears and cessation time and methylation of significant CpG sites

	Packyears^a^		Cessation time^b^	
CpG site	Estimate (se)	*p*-value	Estimate (se)	*p*-value
cg24908166	−0.001 (0.001)	0.24	−0.003 (0.001)	0.02
cg12324353	−0.003 (0.001)	2.3 × 10^−3^	0.002 (0.001)	0.05
cg03725309	−0.002 (0.002)	0.15	0.004 (0.002)	0.02
cg00980784	−0.003 (0.001)	0.01	−0.001 (0.001)	0.69
cg13916835	−0.002 (0.002)	0.14	0.002 (0.002)	0.24
cg09469355	−0.003 (0.001)	0.04	−0.006 (0.001)	6.0 × 10^−5^
cg05603985	−0.002 (0.001)	0.03	0.004 (0.001)	1.2 × 10^−3^
cg04324276	−0.002 (0.001)	0.22	0.002 (0.001)	0.17
cg25468516	−0.001 (0.001)	0.20	0.003 (0.001)	0.06
cg22907952	−0.003 (0.001)	1.8 × 10^−3^	0.002 (0.001)	0.12
cg15474579	−0.004 (0.001)	0.02	0.005 (0.001)	1.5 × 10^−3^
cg20496896	0.002 (0.002)	0.12	−0.003 (0.001)	3.9 × 10^−3^
cg09397246	0.001 (0.002)	0.35	−0.001 (0.002)	0.38
cg26405020	0.002 (0.001)	0.06	−0.002 (0.001)	0.05
cg18236066	−0.002 (0.001)	0.18	0.003 (0.001)	0.04

Bonferroni-corrected threshold 3.3 × 10^−3^

^a^In current smokers, per 10 packyears, adjusted for age, sex, BMI, Houseman estimates, batch effects

^b^In former smokers, per 10 years of smoking cessation, adjusted for age, sex, BMI, Houseman estimates, batch effects

When comparing current to former smokers, the effect estimates were lower and the differences were no longer significant in 10 of the 15 CpG sites (Table 2). This was confirmed in a sensitivity analysis in former smokers, which showed that cessation time was associated with differences in methylation level in three of the identified CpG sites (Table 3).

The two top CpG sites, cg24908166 and cg12324353, were annotated to *TERT* (Table 4). The two CpG sites were positively correlated with each other (*r* = 0.27, *p* < 0.001) and were located within 1 kb from each other in an intron of *TERT*. Two other CpG sites cg05603985 and cg09469355 were located within 1 kb from each other in the first exon and intron of *SKI*. Methylation of these two sites was positively correlated (*r* = 0.57, *p* < 0.001). CpG sites cg09397246 and cg26405020 were located within 1500 base pairs from the transcription start site of *FES.* These sites were within two base pairs from each other and had a positive correlation (*r* = 0.88, *p* < 0.001). The other significant hits were annotated to *SARS*, *GNGT2*, *SMG6*, *TOM1L2*, *SIPA1*, *MRAS*, *CDKN1A*, *LRRC2* and *RPH3A*. The beta-value distributions for all identified CpG sites stratified by the three smoking categories can be found in Additional file 2: Figure S1.

**Table 4:** Characteristics of significant CpG sites

				Average beta-values (SD)		
CpG site	Gene	Position^a^	Placement	Current smokers	Former smokers	Never smokers
cg24908166	TERT	5:1,268,800	Body	0.893 (0.031)	0.900 (0.029)	0.908 (0.024)
cg12324353	TERT	5:1,269,197	Body	0.846 (0.027)	0.851 (0.027)	0.855 (0.024)
cg03725309	SARS	1:109,757,585	Body	0.292 (0.057)	0.297 (0.060)	0.311 (0.063)
cg00980784	GNGT2	17:47,287,577	TSS1500	0.331 (0.048)	0.340 (0.048)	0.341 (0.048)
cg13916835	SMG6	17:2,025,181	Body	0.796 (0.049)	0.812 (0.047)	0.822 (0.039)
cg09469355	SKI	1:2,161,886	Body	0.500 (0.041)	0.516 (0.045)	0.520 (0.040)
cg05603985	SKI	1:2,161,049	First exon	0.347 (0.037)	0.362 (0.038)	0.364 (0.037)
cg04324276	TOM1L2	17:17,817,462	Body	0.502 (0.047)	0.502 (0.046)	0.507 (0.047)
cg25468516	SIPA1	11:65,408,028	5′UTR	0.215 (0.048)	0.218 (0.051)	0.226 (0.054)
cg22907952	MRAS	3:138,121,287	3′UTR	0.806 (0.033)	0.809 (0.034)	0.812 (0.030)
cg15474579	CDKN1A	6:36,753,790	Body	0.706 (0.058)	0.718 (0.052)	0.726 (0.047)
cg20496896	LRRC2	3:46,579,532	Body	0.728 (0.056)	0.727 (0.055)	0.712 (0.056)
cg09397246	FES	15:91,427,361	TSS1500	0.296 (0.066)	0.285 (0.065)	0.286 (0.062)
cg26405020	FES	15:91,427,363	TSS1500	0.464 (0.057)	0.459 (0.059)	0.457 (0.055)
cg18236066	RPH3A	12:113,293,823	Body	0.702 (0.040)	0.709 (0.038)	0.713 (0.041)

TSS1500, 1500 base pairs for transcription start sites

^a^Based on genome build 37

The associations between the 15 CpG sites and mRNA expression of nearby CAD genes are shown in Additional file 1: Table S3. Increased methylation of cg05603985 (*SKI*) was associated with increased expression of cis-eQTL gene *PRKCZ* (estimate = 0.035, *p* = 1.4 × 10^−4^) (Additional file 1: Table S3 and Additional file 2: Figure S2). In the mediation analysis, we did not find a significant proportion of the association between tobacco smoking and expression of *PRKCZ* to be mediated by methylation of cg05603985 (proportion mediated = 0.24, *p* = 0.79). The other CpG sites were not associated with gene expression.

## Discussion

The results of the current study suggest an association between tobacco smoking and DNA methylation of 12 genes suggested to be associated to CAD via GWAS. One of these CpG sites was found to be associated with expression of nearby CAD-related gene *PRKCZ* (protein kinase C, zeta).

We found that the differences in DNA methylation between current and former smokers was lower compared to the difference between current and never smokers in 10 of the 15 CpG sites. This suggests that the effect of tobacco smoking on DNA methylation of these CpG sites is relatively sustained after smoking cessation. To explore the time needed for these CpG sites to recover, we did an analysis on methylation and cessation time and found that cessation time was associated with DNA methylation in 3 of the 15 CpG sites. These results are in line with findings from a recent paper on the relation between tobacco smoking cessation and DNA methylation [6]. We further found that 2 of the 15 CpG sites were associated with cumulative tobacco smoking exposure in current smokers, which is concordant with results from previous studies [6, 7].

The top two CpG sites, cg24908166 and cg12324353, are located within *TERT* (telomerase reverse transcriptase). High levels of *TERT* expression are found in macrophages of human atherosclerotic lesions [10]. Two other CpG sites, cg09397246 and cg26405020, were located near the transcription start site of *FES* (FES proto-oncogene, tyrosine kinase), which has been identified by GWAS to be associated with blood pressure and hypertension [11]. Smoking was further associated with methylation of cg09469355 and cg05603985 within *SKI* (avian sarcoma viral oncogene homolog) which is a repressor of TGF-beta activity. Decreased TGF-beta activity is associated with atherosclerosis development and plaque instability [12, 13]. This could be a plausible pathway through which smoking can increase the occurrence of CAD since smoking has already been associated with decreased plasma levels of TGF-beta and decreased expression of TGF-beta in bronchial cell lines [14, 15].

*PRKCZ* might be the functional gene for rs10797416, the most significant SNP of this locus reported in CAD-GWAS. Rs10797416 has a cis-eQTL effect on *PRKCZ. PRKCZ* is involved in proliferation, differentiation and secretion of almost all cell types including cardiac myocytes and has been associated with cardiomyopathy in chromosome 1p36 deletions [16, 17]. We found that tobacco smoking is associated with decreased methylation of cg05603985, which is in turn associated with decreased expression of *PRKCZ*. We propose that demethylation of cg05603985 might be involved in development of CAD through *PRKCZ* expression, although the causality and mechanism through which this might occur are unclear. Cg05603985 is located in the binding site of transcriptional repressor CTCF [ENCODE:GSM1022677, UW, HCM] [18]. Demethylation of this CTCF binding site facilitates binding of CTCF, resulting in decreased expression of PRKCZ. This is due to the fact that CTCF prevents the binding of enhancers to the promoter of PRKCZ [19].

None of the other CpG sites was associated with the expression of nearby CAD-related genes. The lack of an association does not necessarily mean that methylation of these sites has no effect on expression but could result from an insufficient statistical power. This also applies for the non-significant mediation analysis. Furthermore, mRNA expression is tissue specific and an association may therefore not be found in whole blood. In addition, not all methylation sites in the human genome have an effect on mRNA expression. It could be that these methylation sites function through histone modification or DNA stability which could not be studied in the current work. Finally, it could be that these sites are merely biomarkers of tobacco smoking [5].

The availability of DNA methylation and mRNA expression data from the same samples is a major strength of this study. Therefore, we were able to conduct an in-depth exploration of the association between smoking, DNA methylation and mRNA expression of CAD-related genes. Our study involved methylation and expression data from whole blood samples and not from vascular or lung tissue. This could be a limitation, since methylation and expression might be tissue specific. However, the relationship between smoking and DNA methylation has been confirmed in other tissues including lung tissue [20]. Furthermore, in a study of atherosclerotic tissue, three of our identified genes (PRKCZ, LCCR2 and SMG6) were found to be differentially methylated [21]. This provides further evidence that our findings may be influential in the atherosclerotic pathway. However, this needs further investigation in functional studies. A second limitation is the challenge of gene annotation in GWAS. GWAS locate risk variants for the phenotype under study, but the underlying causal gene might be difficult to designate. To minimize this problem, we limited our analysis to in-gene variants and variants with known cis-eQTL effects. Therefore, the CAD-related genes in our study are more plausible to be actual causal variants for CAD, thus making the results more convincing.

## Conclusions

Our study provides examples of CAD-related genes of which differential methylation is associated with tobacco smoking. Whether or not these genes are in the causal pathway between smoking and coronary artery disease needs further elucidation as well as further efforts in large samples.

## Methods

### Study population

The study was conducted using data from the third cohort of the Rotterdam Study. The design of the Rotterdam Study has previously been described elsewhere [22]. Briefly, all inhabitants from the neighbourhood Ommoord in Rotterdam aged 45 years and over were invited to participate. During the centre visit, 3934 participants were examined between February 2006 and December 2008. We performed the analyses on a random subset of 747 Caucasian subjects from the centre visit. The study was approved by the medical ethics committee at Erasmus University Rotterdam, Rotterdam, the Netherlands, and all examined participants gave written informed consent.

### Data collection

Data on tobacco smoking was collected during home interviews. Participants were asked about past and present cigarette, cigar and pipe smoking behaviour and were then categorized into current, former and never tobacco smokers. Furthermore, information on starting age of smoking, stopping age and time of discontinuation were used to determine packyears in current smokers (median 25.6 packyears, interquartile range 11.4 to 38.0) and cessation time in former smokers (median 21.5 years, interquartile range 10.6 to 32.6). Seven participants had missing smoking status and were therefore excluded from any analysis. During the centre visit, weight and height were measured in standing position wearing normal cloths. All participants had blood samples taken during the visit to quantify DNA methylation, messenger RNA (mRNA) expression levels and other blood measurements.

### DNA methylation data

DNA was extracted from whole peripheral blood (stored in EDTA tubes) by standardized salting out methods. Genome-wide DNA methylation levels were measured using Illumina Human Methylation 450K array [23]. In short, samples (500 ng of DNA per sample) were first bisulfite treated using the Zymo EZ-96 DNA-methylation kit (Zymo Research, Irvine, CA, USA). Next, they were hybridized to the arrays according to the manufacturer’s protocol. The methylation percentage of a CpG site was reported as a beta-value ranging between 0 (no methylation) and 1 (full methylation).

Quality control of the samples was done with Genome Studio. A total number of 16 samples were removed: seven had a sample call rate below 99 %; five had incomplete bisulfite conversion and four had gender swaps. Quality control of the probes was done based on the detection *p*-value calculated with Genome Studio. Probes with a detection *p*-value of more than 0.01 in more than 1 % of the samples were excluded. This resulted in a total set of 474,528 probes which were normalized using the Dasen option of the WateRmelon R-package [24].

### mRNA expression data

Whole-blood was collected (PAXGene Tubes - Becton Dickinson), and total RNA was isolated (PAXGene Blood RNA kits - Qiagen). To ensure a constant high quality of the RNA preparations, all RNA samples were analysed using the Labchip GX (Calliper) according to the manufacturer’s instructions. Samples with an RNA quality score more than 7 were amplified and labelled (Ambion TotalPrep RNA) and hybridized to the Illumina HumanHT12v4 Expression Beadchips as described by the manufacturer’s protocol. Processing of the Rotterdam Study RNA samples was performed at the Genetic Laboratory of Internal Medicine, Erasmus University Medical Centre Rotterdam. The RS-III expression dataset is available at GEO (Gene Expression Omnibus) public repository under the accession GSE33828: 881 samples are available for analysis.

Illumina gene expression data was quantile normalized to the median distribution and subsequently log2 transformed. The probe and sample means were centred to zero. Genes were declared significantly expressed when the detection *p*-values calculated by Genome Studio were less than 0.05 in more than 10 % of all discovery samples, which added to a total number of 21,238 probes. Quality control was done using the eQTL mapping pipeline.

### Statistical analysis

The characteristics of the study population were compared between current and never smokers using IBM SPSS Statistics version 21.0.0.1 (IBM Corp.). The *p*-values were calculated using independent sample *T*-test for continuous variables and chi-square test for dichotomous variables.

Of the 150 SNPs discovered by CARDIoGRAMplusC4D, 96 were located within a gene [1]. In addition, 58 SNPs had known effect on expression of a gene within 1 Mb as found in a large publically available blood cis-eQTL database (FDR < 0.05) [9]. We annotated these SNPs to 85 genes with an in-gene variant and 84 cis-eQTL genes (Additional file 1: Table S1). The methylation probes within and near these CAD-related genes as provided by Illumina were included in the analysis. We excluded probes from the Infinium HD methylation SNP list with a minor allele frequency above 1 % as provided by Illumina, since variations in these SNPs can cause bias in the methylation measurement [25]. We further excluded known cross-reactive probes, since they can introduce bias in the results [26]. A complete list of the probes considered for analysis including reasons for exclusion can be found in Additional file 1: Table S2. The remaining 3669 methylation probes were checked for association with tobacco smoking using a linear mixed model with the LME4 package in R version 3.1.0 with Dasen normalized beta-values of the CpG sites as outcome measure [27]. We first compared current to never smokers and then performed a sensitivity analysis on the identified CpG sites comparing current to former smokers. Covariates were selected based on known association with DNA methylation and different distributions between current and never smokers in our samples. The selected covariates with fixed effects were age, sex and BMI [28–31]. Houseman estimated white blood cell proportions were used as fixed effects to correct for cell mixture distribution [32]. Array number and position on array were added in the model as covariates with random effects to correct for batch effects. We corrected for multiple testing using a robust Bonferroni-corrected *p*-value of 1.4 × 10^−5^ as the threshold for significance (0.05/3669 probes).

To decrease the possibility of confounding in our association, we further adjusted the model in a second analysis for other possible confounders and mediators. This analysis included total cholesterol, triglyceride levels, systolic blood pressure, daily alcohol intake and type 2 diabetes mellitus.

To assess the effect of cumulative exposure to tobacco smoking on DNA methylation, we performed a sensitivity analysis on the identified CpG sites in current smokers with packyears per 10 year as exposure. To assess the effect of discontinuation of tobacco smoking exposure, we performed a sensitivity analysis on the identified CpG sites in former smokers using cessation time per 10 years as exposure. The robust Bonferroni-corrected *p*-value for both analyses was 3.3 × 10^−3^ (0.05/15).

### Functional analysis

Since DNA methylation may have an effect on gene expression, we tested the association between DNA methylation and mRNA expression of nearby CAD-related genes. In the 724 individuals under study, 16 genes out of 26 candidates were found to be expressed in blood. First, we regressed out the Houseman-estimated white blood cell proportions, the erythrocytes and platelet cell counts, fasting state, RNA quality score, plate number, age and sex on the mRNA expression levels using a linear mixed model. We then regressed out the Houseman-estimated white blood cell proportions, age, sex, array number and position on array on the Dasen normalized beta-values of the CpG sites using a linear mixed model. The residuals of the mRNA expression levels and the residuals of the Dasen normalized beta-values of the CpG sites were checked for association using a linear regression model. We analysed the associations between the 15 methylation sites and 16 nearby CAD genes. Additional file 1: Table S3 indicates the 35 CpG-gene combinations that were tested. The robust Bonferroni-corrected *p*-value threshold for a significant association was 1.4 × 10^−3^ (0.05/35 tests). Significant associations were reviewed in a mediation analysis with current versus never smoking as exposure, Dasen normalized beta-values of the CpG site as potential mediator and mRNA expression levels as outcome using the mediation package in R [33]. The method applied in the mediation package calculates the proportion of the association between tobacco smoking and expression that is mediated by methylation. This is done by comparing the effect estimates of tobacco smoking on expression in subjects with different levels of methylation.

## Additional files

Additional file 1: Tables S1 to S3
**Table S1.** List of CARDIoGRAMplusC4D SNPs and annotated in-gene variants and cis-eQTL genes. **Table S2.** List of considered CpG sites in/near CAD and cis-eQTL genes with exclusion reasons. **Table S3.** Association between methylation of significant CpG sites and expression of nearby CAD-related genes. Additional file 2: Figure S1 and S2
**Figure S1.** Beta-value distributions of significant CpG sites per smoking category. **Figure S2**. Relation between methylation of cg05603985 and expression of *PRKCZ*.

## Abbreviations

CAD: coronary artery disease
CpG: cytosine-(phosphate)-guanine
eQTL: expression quantitative trait loci
GWAS: genome-wide association study
mRNA: messenger RNA
SNP: single nucleotide polymorphism

## References

[1] CARDIoGRAMplusC4D Consortium. Large-scale association analysis identifies new risk loci for coronary artery disease. Nat Genet. 2013;45(1):25–33. doi:10.1038/ng.2480. 10.1038/ng.2480 PMC3679547 23202125

[2] Cheng S, Claggett B, Correia AW, Shah AM, Gupta DK, Skali H (2014). Temporal trends in the population attributable risk for cardiovascular disease: the atherosclerosis risk in communities study. Circulation.

[3] Vecoli C, Adlerstein D, Shehi E, Bigazzi F, Sampietro T, Foffa I (2014). Genetic score based on high-risk genetic polymorphisms and early onset of ischemic heart disease in an Italian cohort of ischemic patients. Thromb Res.

[4] Niemiec P, Nowak T, Iwanicki T, Krauze J, Gorczynska-Kosiorz S, Grzeszczak W (2014). The 930A>G polymorphism of the CYBA gene is associated with premature coronary artery disease: a case–control study and gene-risk factors interactions. Mol Biol Rep.

[5] Jones PA (2012). Functions of DNA methylation: islands, start sites, gene bodies and beyond. Nat Rev Genet.

[6] Zeilinger S, Kuhnel B, Klopp N, Baurecht H, Kleinschmidt A, Gieger C (2013). Tobacco smoking leads to extensive genome-wide changes in DNA methylation. PLoS One.

[7] Breitling LP, Yang R, Korn B, Burwinkel B, Brenner H (2011). Tobacco-smoking-related differential DNA methylation: 27K discovery and replication. Am J Hum Genet.

[8] Joubert BR, Haberg SE, Nilsen RM, Wang X, Vollset SE, Murphy SK (2012). 450K epigenome-wide scan identifies differential DNA methylation in newborns related to maternal smoking during pregnancy. Environ Health Perspect.

[9] Westra H-J, Peters MJ, Esko T, Yaghootkar H, Schurmann C, Kettunen J (2013). Systematic identification of trans eQTLs as putative drivers of known disease associations. Nat Genet.

[10] Gizard F, Heywood EB, Findeisen HM, Zhao Y, Jones KL, Cudejko C (2011). Telomerase activation in atherosclerosis and induction of telomerase reverse transcriptase expression by inflammatory stimuli in macrophages. Arterioscler Thromb Vasc Biol.

[11] International Consortium for Blood Pressure Genome-Wide Association Studies (2011). Genetic variants in novel pathways influence blood pressure and cardiovascular disease risk. Nature..

[12] Lebastchi AH, Qin L, Khan SF, Zhou J, Geirsson A, Kim RW (2011). Activation of human vascular cells decreases their expression of transforming growth factor-beta. Atherosclerosis.

[13] Mallat Z, Gojova A, Marchiol-Fournigault C, Esposito B, Kamate C, Merval R (2001). Inhibition of transforming growth factor-beta signaling accelerates atherosclerosis and induces an unstable plaque phenotype in mice. Circ Res.

[14] Kamio K, Ishii T, Motegi T, Hattori K, Kusunoki Y, Azuma A (2013). Decreased serum transforming growth factor-beta1 concentration with aging is associated with the severity of emphysema in chronic obstructive pulmonary disease. Geriatr Gerontol Int.

[15] Samanta D, Gonzalez AL, Nagathihalli N, Ye F, Carbone DP, Datta PK (2012). Smoking attenuates transforming growth factor-beta-mediated tumor suppression function through downregulation of Smad3 in lung cancer. Cancer Prev Res (Phila).

[16] Shimada S, Shimojima K, Okamoto N, Sangu N, Hirasawa K, Matsuo M, et al. Microarray analysis of 50 patients reveals the critical chromosomal regions responsible for 1p36 deletion syndrome-related complications. Brain Dev. 2014. doi: 10.1016/j.braindev.2014.08.002. 10.1016/j.braindev.2014.08.002 25172301

[17] Zaveri HP, Beck TF, Hernandez-Garcia A, Shelly KE, Montgomery T, van Haeringen A (2014). Identification of critical regions and candidate genes for cardiovascular malformations and cardiomyopathy associated with deletions of chromosome 1p36. PLoS One.

[18] Encode Project Consortium (2012). An integrated encyclopedia of DNA elements in the human genome. Nature..

[19] Bell AC, Felsenfeld G (2000). Methylation of a CTCF-dependent boundary controls imprinted expression of the Igf2 gene. Nature.

[20] Shenker NS, Polidoro S, van Veldhoven K, Sacerdote C, Ricceri F, Birrell MA (2013). Epigenome-wide association study in the European Prospective Investigation into Cancer and Nutrition (EPIC-Turin) identifies novel genetic loci associated with smoking. Hum Mol Genet.

[21] Zaina S, Heyn H, Carmona FJ, Varol N, Sayols S, Condom E (2014). DNA methylation map of human atherosclerosis. Circ Cardiovasc Genet.

[22] Hofman A, Darwish Murad S, van Duijn CM, Franco OH, Goedegebure A, Ikram MA (2013). The Rotterdam Study: 2014 objectives and design update. Eur J Epidemiol.

[23] Sandoval J, Heyn H, Moran S, Serra-Musach J, Pujana MA, Bibikova M (2011). Validation of a DNA methylation microarray for 450,000 CpG sites in the human genome. Epigenetics.

[24] Pidsley R, Wong CCY, Volta M, Lunnon K, Mill J, Schalkwyk LC (2013). A data-driven approach to preprocessing Illumina 450K methylation array data. BMC Genomics..

[25] Zhi D, Aslibekyan S, Irvin MR, Claas SA, Borecki IB, Ordovas JM (2013). SNPs located at CpG sites modulate genome-epigenome interaction. Epigenetics.

[26] Chen YA, Lemire M, Choufani S, Butcher DT, Grafodatskaya D, Zanke BW (2013). Discovery of cross-reactive probes and polymorphic CpGs in the Illumina Infinium Human Methylation450 microarray. Epigenetics.

[27] R Core Team (2014). R: a language and environment for statistical computing.

[28] Koestler DC, Christensen B, Karagas MR, Marsit CJ, Langevin SM, Kelsey KT (2013). Blood-based profiles of DNA methylation predict the underlying distribution of cell types: a validation analysis. Epigenetics.

[29] Dick KJ, Nelson CP, Tsaprouni L, Sandling JK, Aissi D, Wahl S et al. DNA methylation and body-mass index: a genome-wide analysis. Lancet. 2014. doi:10.1016/S0140-6736(13)62674-4. 10.1016/S0140-6736(13)62674-4 24630777

[30] Florath I, Butterbach K, Muller H, Bewerunge-Hudler M, Brenner H (2014). Cross-sectional and longitudinal changes in DNA methylation with age: an epigenome-wide analysis revealing over 60 novel age-associated CpG sites. Hum Mol Genet.

[31] Zhang FF, Cardarelli R, Carroll J, Fulda KG, Kaur M, Gonzalez K (2011). Significant differences in global genomic DNA methylation by gender and race/ethnicity in peripheral blood. Epigenetics.

[32] Houseman EA, Accomando WP, Koestler DC, Christensen BC, Marsit CJ, Nelson HH (2012). DNA methylation arrays as surrogate measures of cell mixture distribution. BMC Bioinformatics..

[33] Tingley DYT, Keele L, Imai K (2014). Mediation: R package for causal mediation analysis. J Statistical Softw..

