# Supplementary material for: Tobacco smoking is associated with methylation of genes related to coronary artery disease
Source: Clin Epigenetics. 2015 May 14;7(1):54. doi: 10.1186/s13148-015-0088-y (PMC4443552; doi:10.1186/s13148-015-0088-y)
Supplement: Additional file 2: Figure S1 and S2 — Figure S1. Beta-value distributions of significant CpG sites per smoking category. Figure S2. Relation between methylation of cg05603985 and expression of PRKCZ. [file 13148_2015_88_MOESM2_ESM.pdf]

Additional file 2

Figure S1 – Beta-value distributions of significant CpG sites per smoking category

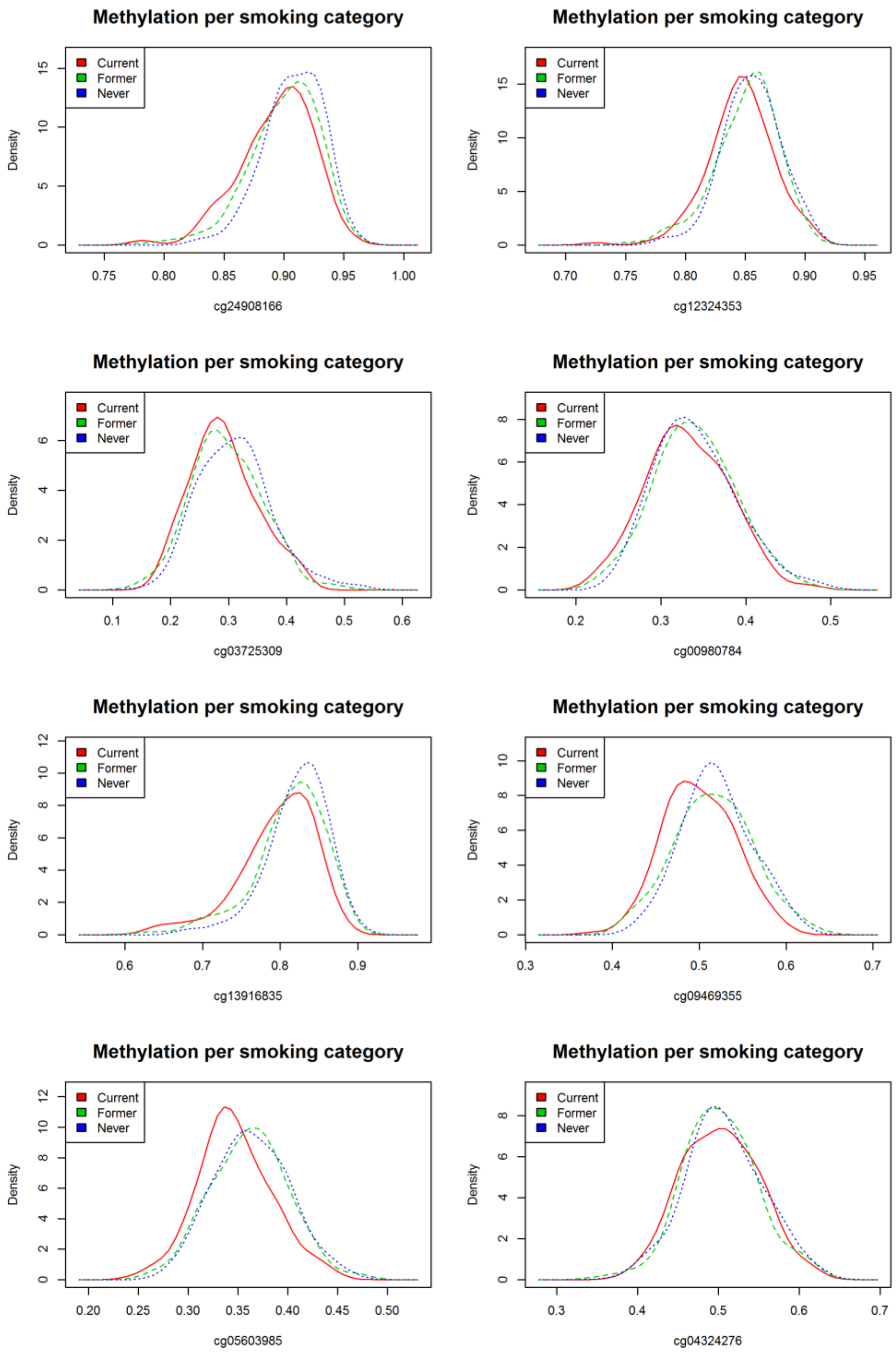

Kernel density plots of the methylation beta-values of the significant CpG sites stratified by smoking category.

Figure S1 Continued – Beta-value distributions of significant CpG sites per smoking category

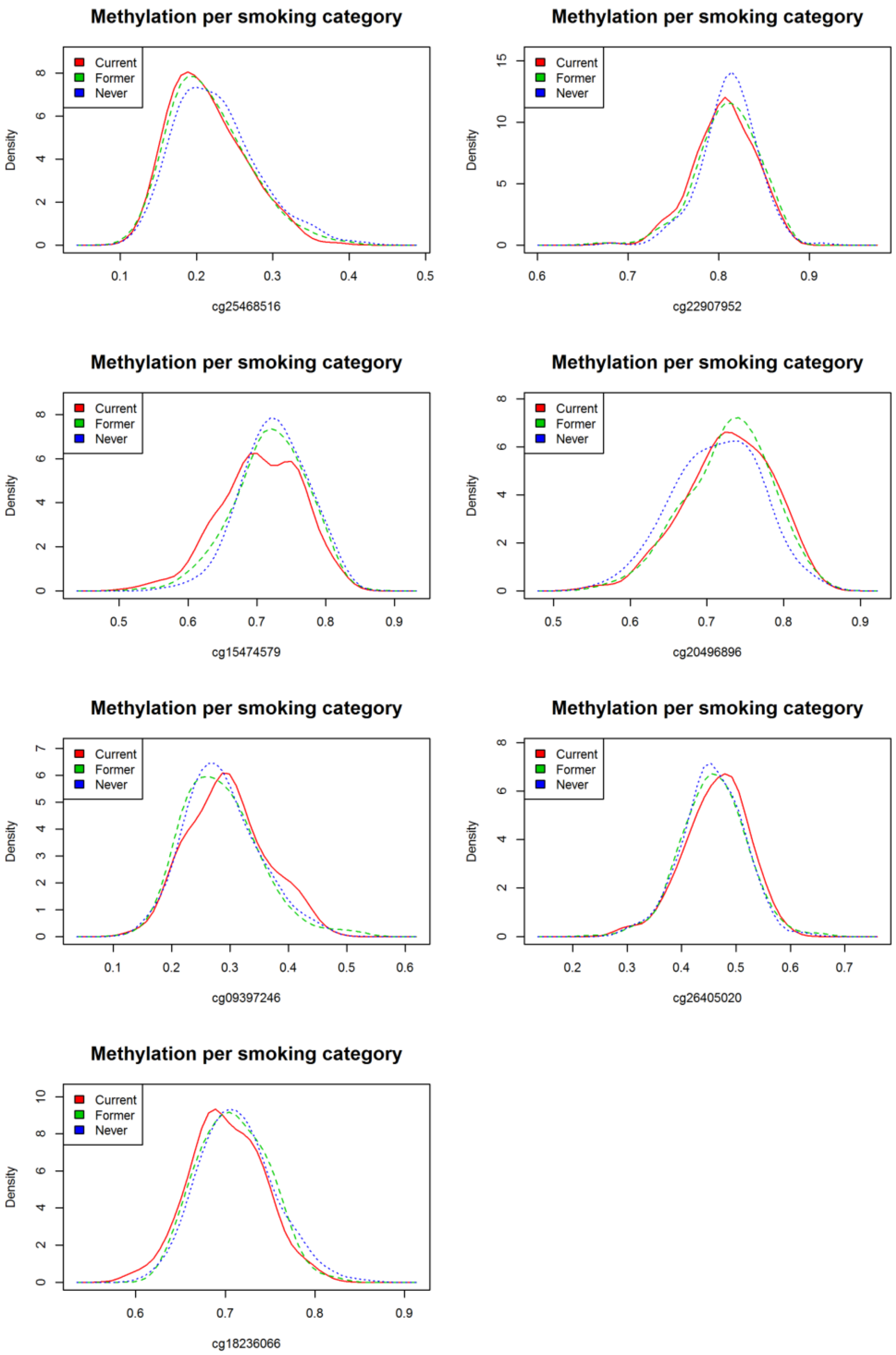

Kernel density plots of the methylation beta-values the significant CpG sites stratified by smoking category.

Figure S2 - Relation between methylation of cg05603985 and expression of *PRKCZ*

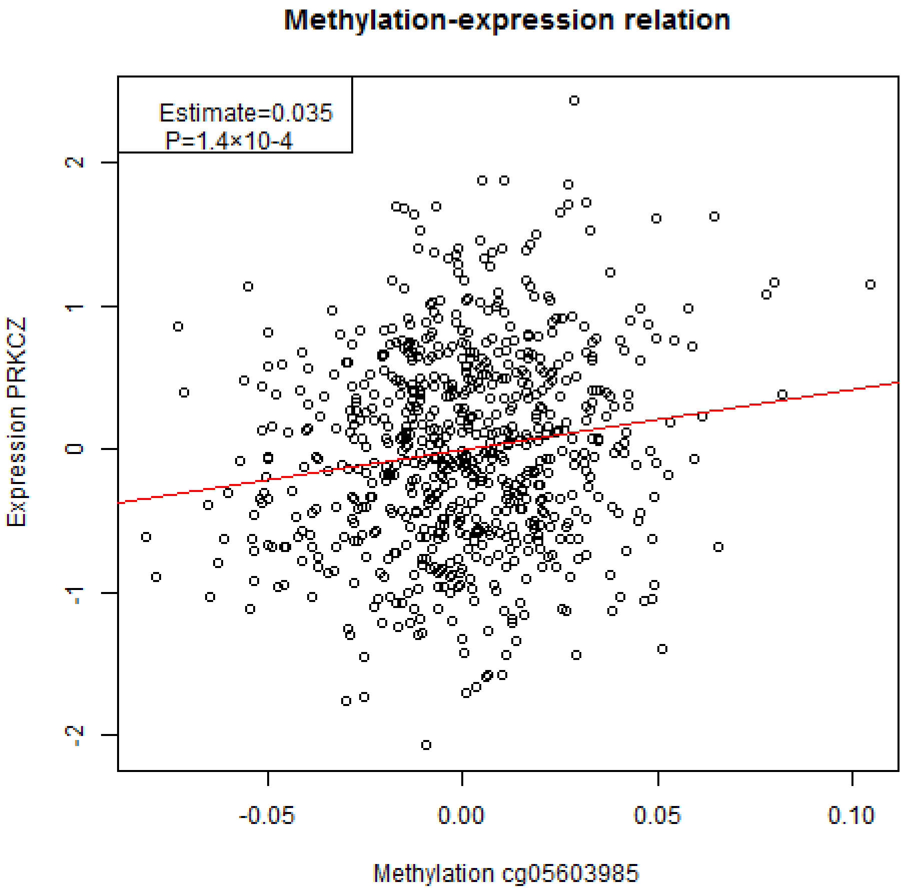

PRKCZ expression is quantile-normalized to the median distribution and subsequently log2-transformed, after which residuals were created by regressing out age, sex, batch effects, houseman estimated white blood cell proportions, erythrocytes and platelet cell counts, fasting state and RNA quality score. Cg05603985 methylation are residual Dasen normalized beta-values after regressing out age, sex, houseman estimated white blood cell proportions and batch effects. Estimate is change in residual expression per percentage residual methylation increase.
